# Using the analytic hierarchy process to assess the severity of psychopathological states

**DOI:** 10.1192/j.eurpsy.2022.1951

**Published:** 2022-09-01

**Authors:** V. Mitikhin, T. Solokhina, G. Tiumenkova, M. Kuzminova

**Affiliations:** FSBSI “Mental Health Research Centre”, Department Of Mental Health Services, Moscow, Russian Federation

**Keywords:** psychopalogy, algorithm, analytic hierarchy process, severity

## Abstract

**Introduction:**

Currently, there are known problems of assessing the severity of psychopathological states based on psychometric (rank) scales [1]. The main problem: ranks are non-numeric information that does not allow the simplest mathematical operations (summation, average) [2] and, as a result, the impossibility of constructing correct models for evaluating states

**Objectives:**

Development of algorithms for processing initial rank information about the severity of psychopathological states in order to obtain results in numerical form based on the Analytic Hierarchy Process (AHP) [2]

**Methods:**

Clinical, statistical, algorithms of the AHP.

**Results:**

The problems of assessing the patient’s states are multicriteria. They are solved within the framework of AHP by constructing numerical intensity scales when measuring the dimensions of disorders. This means a correct transition from the rank scale to the scale of relations, in which the estimates are numbers that allow any mathematical operations. The implementation of AHP procedures is based on the application of the AHP normative approach [2], which uses expert comparisons of ratings of the rank scale.

**Conclusions:**

The fundamental difference between the results based on AHP and rank results is due to the fact that numerical estimates of the severity of states are obtained, which can be used for any mathematical processing and the construction of correct models of communication and prediction of the state of patients from many factors, taking into account their weight. References: 1. Zimmerman M., Morgan T.A., Stanton K. *World Psychiatry.* 2018;17: 258–275. 2. Mitikhin, V.G., Solokhina, T.A. *S.S. Korsakov Journal of Neurology and Psychiatry.* 2019; 119(2): 49-54.

**Disclosure:**

No significant relationships.

